# Editorial: The complex cross-kingdom interactions between plant, mycorrhizal fungi and bacteria: current status and emerging opportunities

**DOI:** 10.3389/fmicb.2025.1721753

**Published:** 2025-11-03

**Authors:** Xiaoli Yu, Mingfei Chen, Yuguo Yang, Huang Yu, Ruiwen Hu

**Affiliations:** ^1^Marine Synthetic Ecology Research Center, Southern Marine Science and Engineering Guangdong Laboratory (Zhuhai), School of Marine Sciences, Sun Yat-sen University, Zhuhai, China; ^2^Lawrence Berkeley National Laboratory, Department of Ecology, Earth & Environmental Sciences Area, Berkeley, CA, United States; ^3^Key Discipline Laboratory for National Defense for Biotechnology in Uranium Mining and Hydrometallurgy, University of South China, Hengyang, China; ^4^Environmental Genomics and Systems Biology Division, Lawrence Berkeley National Laboratory, Berkeley, CA, United States

**Keywords:** cross-kingdom interactions, plant, mycorrhizal fungi, bacteria, synthetic microbial communities

Plants establish multifaceted interactions with mycorrhizal fungi and bacteria across distinct ecological compartments, including the rhizosphere, endosphere, and phyllosphere. These interactions are shaped by plant species, genetics, metabolites, and environmental factors, and are increasingly recognized as critical drivers of plant health, nutrient cycling, and ecosystem resilience (Trivedi et al., 2022; Compant et al., 2025). Elucidating the mechanisms underlying these cross-kingdom interactions is essential for harnessing microbial functions to enhance plant tolerance to environmental stresses, improve agricultural productivity, and mitigate the impacts of climate change. Recent research has highlighted several major mechanisms governing plant-fungi-bacteria interactions, including chemical signaling via root exudates and volatile organic compounds, nutrient exchange and recycling among partners, and cooperative defense against pathogens (Sasse et al., 2018; Camargo et al., 2023; Jansson et al., 2023; Razo-Belmán et al., 2023; Yang et al., 2024). Root exudates (e.g., flavonoids, siderophores, terpenes) are now recognized as key regulators of plant-microbe communication, shaping microbial recruitment and influencing plant performance (Fan et al., 2025). However, the majority of root exudate compounds remain structurally and functionally uncharacterized, limiting a comprehensive understanding of their ecological and evolutionary significance. Symbioses between plants and arbuscular mycorrhizal fungi (AMF) provide another key example of microbe-mediated nutrient exchange, particularly in nitrogen and phosphorus acquisition (Bennett and Groten, 2022). Beyond the rhizosphere, the hyphosphere (i.e., the microenvironment surrounding AMF hyphae), provides an interface for plant-AMF-bacteria interactions (Duan et al., 2024). Yet, the ecological roles of hyphosphere-associated bacteria and their contribution to AMF functionality remain poorly understood. Traditional approaches to studying plant-microbe interactions have often relied on multi-omics analyses or the characterization of individual microbial strains. By contrast, synthetic ecology offers an alternative framework for studying these interactions by enabling researchers to investigate the complex dynamics and functional contributions of specific microbial consortia in relation to plant health and growth. For example, synthetic microbial communities (SynComs) composed of aluminum-tolerant bacterial strains isolated from the rice rhizosphere have been shown to alleviate aluminum toxicity and enhance rice yield (Liu et al., 2023). Despite these advances, major challenges remain for SynCom-based strategies, including the rational selection of functionally complementary strains, optimization of community composition, and prediction of long-term ecological stability under field conditions. Overall, substantial progress has been made in understanding the mechanistic bases of plant–microbe interactions. Nevertheless, critical knowledge gaps remain, particularly regarding the chemical diversity of root exudates, the roles of bacteria in the hyphosphere, and the ecological predictability of engineered SynComs.

To address these knowledge gaps, our Research Topic titled “*The Complex Cross-Kingdom Interactions Between Plant, Mycorrhizal Fungi and Bacteria: Current Status and Emerging Opportunities*” presents seven publications that provide valuable insights into the role of mycorrhizal fungi and bacteria in shaping plant health and development, as well as into their interaction mechanisms. The rhizosphere microbiome is a hotspot for interactions between plants and microbes, and understanding its composition and function is crucial for plant development and health. Sarsaiya et al. provided a comprehensive overview of *Dendrobium* rhizosphere microbiome, highlighting its roles in supporting plant growth, health and resilience. They also underscored the importance of signaling molecules in driving plant-microbe interactions, emphasizing their potential applications in designing SynComs and microbiome engineering for sustainable agriculture. Plant-microbe interactions play a crucial role in regulating ecosystem functions, including carbon emissions. He et al. demonstrated that mangrove species significantly alter sediment physicochemical properties and microbial communities, subsequently influencing carbon fluxes. This study underscores the importance of plant-sediment-microbe interactions in the mangrove carbon cycle and offers valuable insights for informed mangrove management strategies. Fungi exhibit diverse interactions with plants, ranging from mutualistic to pathogenic. AMF promote plant performance by improving nutrient uptake through their hyphal network and modulating phytohormone signaling pathways (Wang et al., 2023). Zhuang et al. found that AMF can optimize nutrient cycling, improve soil structure, enhance plant physiological metabolism, and alter soil microbial community composition in an areca/vanilla intercropping system under nitrogen-limited conditions. This study highlights the potential of AMF in agricultural management, particularly in reducing fertilizer dependency. Conversely, pathogenic fungi greatly contribute to global crop yield losses, making it essential to understand their pathogenic mechanisms for the development of effective mitigation strategies. The hyphosphere represents a unique microenvironment where microbes interact intensively, potentially shaping fungal pathogenicity. Antony-Babu et al. characterized the bacterial community in the hyphosphere of the cotton pathogen *Fusarium oxysporum*. By isolating 10 bacterial strains from hyphosphere, they identified a stable, *Pseudomonas*-dominated community that enhances fungal fitness and virulence. The authors suggest that the hyphosphere-pathobiome is a potential target for disease management. Microbes also support plants in coping with environmental stresses. Zhu et al. investigated the effects of manganese stress on the rhizosphere soil physicochemical properties and microbial communities of sugarcane in acidic soils. They found that manganese stress suppressed sugarcane growth by inhibiting nitrogen uptake and reducing nitrogen-fixing taxa, effects that could be alleviated through organic fertilization. Similarly, Luo et al. investigated organic fertilizer-mediated remediation of antimony-contaminated soils and found that fertilization enriched microbial groups involved in Sb(III) oxidation, nitrogen fixation, and nutrient solubilization, thereby enhancing Sb(III) detoxification and alleviating plant stress. Synthetic microbial communities (SynComs) further provide opportunities for sustainable agriculture and environmental remediation. Yadav et al. integrated network analysis and cultivation-based methods to construct a SynCom containing 15 strains from *Brachypodium distachyon* rhizosphere. This SynCom promoted plant growth under drought stress by facilitating osmoprotectant synthesis and regulating sodium-potassium transport, highlighting its potential to improve plant resilience to environmental challenges.

These studies collectively highlight the intricate and essential role of cross-kingdom interactions in regulating plant health and ecosystem processes, while also offering new perspectives for advancing microbiome engineering toward sustainable agriculture. Building on these insights, we propose several priorities for future research: First, molecular and systems-level approaches should be systematically employed to unravel the molecular mechanisms underlying plant-mycorrhizal fungi-bacteria interactions, with particular emphasis on signaling cascades and metabolite exchange that mediate inter-kingdom communication. Second, it is critical to determine how such plant-microbe associations respond to global change drivers, including climate variability, environmental pollution, and diverse environmental stresses. Finally, long-term experimental and field-based approaches are needed to evaluate microbiome engineering strategies and to enable robust assessments of their impacts on plant performance, ecosystem stability, and resilience.
